# Relationship between ZHX2 Expression and VHL Gene Alteration in VHL-associated and Sporadic Hemangioblastomas of the Central Nervous System

**DOI:** 10.15586/jkcvhl.v11i4.355

**Published:** 2024-12-31

**Authors:** Hiroshi Kanno, Kimihiro Nakahara, Sachiko Yamada, Satoshi Fujii, Hidetoshi Murata, Tetsuya Yamamoto, Hisashi Hasumi, Masahiro Yao

**Affiliations:** 1 Department of Neurosurgery, Yokohama City University Graduate School of Medicine, Yokohama, Japan;; 2 Department of Neurosurgery, International University of Health and Welfare, Narita, Japan;; 3 Department of Neurosurgery, Asahi Hospital, Tokyo, Japan;; 4 Department of Neurosurgery, St. Marianna University School of Medicine, Kawasaki, Japan;; 5 Department of Urology, Yokohama City University Graduate School of Medicine, Yokohama, Japan

**Keywords:** central nervous system hemangioblastoma, gene alteration, VHL, ZHX2

## Abstract

Central nervous system hemangioblastoma (CNS-HB) is the most common manifestation of von Hippel–Lindau disease (VHL). The main axis of the CNS-HB pathway is the VHL-HIF signaling pathway. Recently, we proposed an alternative VHL-JAK-STAT pathway in CNS-HB. In contrast, the VHL substrate transcription factor zinc fingers and homeoboxes 2 (ZHX2) have been identified as the oncogenic drivers in VHL-deficient clear cell renal cell carcinoma (RCC). However, ZHX2 expression in CNS-HB has not been previously reported. Furthermore, the VHL-ZXH2-NF-κB signaling pathway in CNS-HB remains unresolved. In this study, we aimed to investigate ZHX2 expression and VHL gene alteration in CNS-HB and propose the role of ZHX2 in CNS-HB. Using the MACS method, Scl+ hemangioblastoma-like cells were isolated from multipotent nestin-expressing stem cells. The ubiquitination of ZHX2 in these cells and the immunoprecipitation between ZHX2 and VHL were investigated. In addition, the VHL genes of patients with hemangioblastoma were analyzed. ZHX2 expression in CNS-HB tissues was examined by immunohistochemistry and western blotting. In addition, VHL gene mutations in CNS-HB were analyzed by sequencing. The association between ZHX2 expression and VHL gene mutation was analyzed. ZHX2 was ubiquitinated in Scl+hemangioblastoma-like cells after the transfer of the VHL expression vector into these cells. ZHX2 expression in these cells was well detected before transfer but disappeared after the transfer. ZHX2 expression was detected in 18 of the 21 CNS-HB tissues by immunoblotting and/or immunohistochemistry. Sporadic CNS-HB showed weak expression, whereas VHL-related CNS-HB showed moderate or strong expression. In particular, CNS-HB with severe VHL gene mutations, including large deletions, showed strong or moderate ZHX2 expression. The association between VHL gene mutation and ZHX2 expression revealed a significant correlation between VHL gene alteration severity and the level of immunoblotting (P < 0.05). In conclusion, the severity of VHL gene alteration correlates with the level of ZHX2 expression. ZHX2 is predominantly expressed in CNS-HB, especially in VHL-related cases with severe VHL gene alterations, suggesting a potential role in tumorigenesis and proliferation of CNS-HB.

## Introduction

Central nervous system hemangioblastoma (CNS-HB) is the predominant manifestation of von Hippel–Lindau disease (VHL). Both VHL-associated and sporadic CNS-HB arise following inactivation of the VHL gene, the gene responsible for VHL (1, 2). Alterations in the VHL gene are consistently identified in VHL-related CNS-HB tissues, whereas sporadic CNS-HBs rarely show such alterations (3, 4). The VHL gene consists of α and β domains, and mutations are predominantly concentrated in these domains. The β-domain contains the binding site for elongin C (5), while the β-domain contains a binding site for the hypoxia-inducible factor (HIF) (6). Formation of the VHL/E3 complex involves the recruitment of elongin C, elongin B, cullin2, Rbx1, and E2 by VHL. VHL then binds to HIF, leading to HIF ubiquitination and subsequent degradation by the 26S proteasome. The VHL-HIF signaling pathway plays a pivotal role in CNS-HB angiogenesis (7). CNS-HBs, characterized by high vascularity, exhibit vascular endothelial growth factor (VEGF) production. Erythropoietin overproduction has been associated with polycythemia and intratumoral extramedullary hematopoiesis in CNS-HB (8). Both VEGF and erythropoietin (EPO) are upregulated by HIF, and coexpression of VEGF and EPO is observed in CNS-HB hemangioblastomas (9). Furthermore, VHL protein (pVHL)-defective cells have elevated levels of hypoxia-inducible mRNAs, including VEGF mRNA, a defect that can be corrected by restoring pVHL function (10). In addition, our recent research suggests that the VHL-JAK-STAT pathway serves as an alternative signaling pathway that influences cell proliferation, maintenance of stem cell characteristics, and angiogenesis in hemangioblastoma (11).

Previously, zinc finger and homeobox 2 (ZHX2) were reported to be tumor suppressors in hepatocellular carcinoma (HCC) and lymphoma (12, 13). In addition, the mRNA levels of its related family members, ZHX1 and ZHX3, have been associated with the pathological stage of clear cell renal cell carcinoma (ccRCC) (14). Thus, ZHX2 was found to be an oncogenic driver in VHL-deficient ccRCC. Tumor cells from ccRCC patients with VHL loss-of-function mutations typically show increased abundance and nuclear localization of ZHX2. Functionally, depletion of ZHX2 inhibits the growth of VHL-deficient ccRCC cells in vitro and in vivo (15). Although it has been speculated that ZHX2 plays an important role in CNS-HB, the expression of ZHX2 in CNS-HB has not been investigated. The VHL substrate transcription factor zinc fingers and homeoboxes 2 (ZHX2) have been identified as an oncogenic driver in VHL-deficient clear cell renal cell carcinoma (RCC). However, ZHX2 expression in CNS-HB has not been previously reported. Furthermore, the VHL-ZXH2-NF-κB signaling pathway in CNS-HB remains unresolved. In this study, we report ZHX2 expression and VHL gene alterations in CNS-HB and discuss the role of ZHX2 in CNS-HB.

## Materials and Methods

### Blood samples and tumor tissues

Blood samples and tumor tissues were obtained from patients diagnosed with CNS-HB at Yokohama City University Hospital and its affiliated hospitals. All samples were snap-frozen with liquid nitrogen or stored at −80°C, and formalin fixation was performed before nucleic acid or protein extraction. A portion of each sample was further fixed in formalin and embedded in paraffin for pathological analysis. Written informed consent was obtained from patients for germline, somatic gene, and protein expression analyses, and the study protocol was approved by the Institutional Ethics Committee.

### Sequencing analysis and RQ-PCR

DNA was extracted from peripheral blood samples of patients with VHL and CNS-HB tissues based on a previously described method (1). Polymerase chain reaction (PCR) products were directly sequenced according to the manufacturer’s instructions. Sequencing reactions of PCR-amplified DNA were performed on an automated sequencer (ABI PRISM 310 Genetic Analyzer; Perkin-Elmer Japan) using the same primers as previously described (16). In addition, we performed RQ-PCR for VHL deletion detection as described in a previous report (17). RQ-PCR primers and TaqMan probes (Nippon EGT Co., Ltd., Tokyo, Japan) were designed for the three VHL-encoding exons and/or surrounding intronic regions.

### Scl+ haemangioblastoma-like cells

This study used Scl+ hemangioblastoma-like cells derived from multipotent nestin-expressing stem cells. As described previously (10), Scl+ hemangioblastoma-like cells were isolated as follows: Multipotent nestin-expressing stem cells (MNSCs) were previously isolated by us from hair follicles of elderly humans (18). These cells were cultured in growth medium consisting of DMEM/F12 (1:1; Gibco, Grand Island, NY, USA) containing 2% B27 supplement (Gibco), 20 ng/mL epidermal growth factor (EGF; Upstate Biotechnology, Lake Placid, NY, USA), and 40 ng/mL bFGF (PeproTech EC Ltd. London, UK) in a humidified incubator at 37°C with 5% CO_2_. Our previous study showed that MNSCs expressed high levels of nestin, CD34, and fibronectin, and low levels of NGFRp75 and keratin 15. In addition, MNSCs could spontaneously differentiate into various cell types, including astrocytes, neurons and smooth muscle cells (18). In the next step, Scl+ cells derived from MNSCs (Scl+ MNSCs-derived cells) were isolated using an autoMACS™ Pro separator (Miltenyi Biotec, Bergisch Gladbach, Germany). The immunocytochemical study of Scl+ MNSC-derived cells showed that they were not only Scl positive, but also Flk-1 and Tie-1 positive. However, MAP-2 and GFAP were negative. This result suggested that Scl+ MNSC-derived cells are similar to embryonic hemangioblasts, which are thought to be the origin of hemangioblatomas. Scl+ MNSC-derived cells, that is, Scl+embryonic hemangioblast-like cells, were then used as hemangioblatoma-like cells for further studies.

### VHL-expressing adenovirus vector

The VHL-expressing adenovirus vector was prepared as previously described (18). Briefly, an adenovirus vector encoding human VHL (VHL54-213 amino acids) was generated using the cosmid vector pAxCAwt. As a control vector, the green fluorescent protein (GFP) vector was obtained from the Riken Gene Bank (Saitama, Japan) and generated using the cosmid vector pAxCAwt. For adenovirus infection, Scl+MNSC-derived cells were incubated for 1 h with 5 μL of the virus solution diluted to 1 × 10^7^ plaque-forming units per milliliter in DMEM/F12 medium containing 5% fetal calf serum at a multiplicity of infection of 10, a condition sufficient for nearly 100% infection of the cells. The VHL-expressing adenoviral vector was transferred to the cells after dissociation from the sphere into single cells. Two days after transfection of the cells, the cultured cells were fixed on coverslips for immunocytochemical study and protein was extracted for immunoprecipitation or Western blot analysis.

### Immunoprecipitation

Cultured cells (5 × 10^6^) were washed with ice-cold PBS and lysed in RIPA lysis buffer (Upstate, Charlottesville, VA). Total protein was extracted from the cells and the total protein concentration of each lysate was adjusted to 1 mg/mL. The lysates were immunoprecipitated with anti-VHL antibody (Santa Cruz, San Diego, CA, USA) using μMACS Protein A microbeads (Miltenyi Biotec, Bergisch Gladbach, Germany). Immunoprecipitates were analyzed by SDS/gradient polyacrylamide gel and Western blotting with anti-ZHX2 antibody diluted 1:500. Immunolabeled bands were detected using enhanced chemiluminescence reagents (Amersham Biosciences Corp., Piscataway, NJ, USA). Images were analyzed using LAS-1000 (Fujifilm, Tokyo, Japan), and the density of the bands was determined using Image Gauge software (Fujifilm, Tokyo, Japan).

### Ubiquitination assay

As previously described (11), the ubiquitination reaction was performed with the addition of 10 mM ubiquitin (Sigma-Aldrich), 2 mM ubiquitin-activating enzyme solution (E1; Enzo Life Sciences, Inc., Farmingdale, NY, USA), and 2 mM ubiquitin-conjugating enzyme solution (E2; Enzo Life Sciences, Inc.) and VHL-expressing adenovirus vector or control vector. After incubation for 4 h, cultured cells (1 × 10^6^) were washed with ice-cold PBS and lysed in RIPA lysis buffer (Upstate, Charlottesville, VA, USA), total protein was extracted from the cells, and the lysates were immunoprecipitated with anti-ZHX2 antibody (Santa Cruz, San Diego, CA, USA) using Protein A/G Sepharose (Abcam, Cambridge, MA, USA). The immunoprecipitates were supplemented with 1 mM PMSF and the protease inhibitor cocktail. After 2 h, each sample was separated by SDS-PAGE and transferred electrophoretically to nitrocellulose filters. Western blots were probed with anti-ubiquitin antibody (Sigma-Aldrich) followed by horseradish peroxidase-conjugated secondary antibodies (Amersham). Protein bands were detected using a chemiluminescence detection system (Amersham). Images were analyzed using LAS-1000 (Fujifilm, Tokyo, Japan).

### Immunohistochemistry

Paraffin sections of 14 μm thickness were deparaffinized and rehydrated before processing for hematoxylin and eosin (H&E) staining and immunohistochemistry. For immunostaining, nonspecific antigenic proteins were blocked with 10% normal donkey serum, followed by incubation with primary antibody against ZHX2 (1:200; Proteintech Group Inc., London, UK) at 4°C overnight. The sections were then rinsed and incubated with a secondary antibody, biotin-conjugated anti-rabbit IgG (1:200; Vector Laboratory, Inc., Newark, CA, USA). This was followed by incubation with horseradish peroxidase (HRP)-conjugated biotin and avidin complexes (1:200; Vector Laboratory, Inc.). Visualization was achieved by development with 3,3’-diaminobenzidine. Counterstaining was performed with hematoxylin. Finally, the sections were coverslipped and examined under a microscope.

### Western blotting

Proteins were extracted from frozen tissues and formalin-fixed, paraffin-embedded samples using the MINUTE Total Protein Extraction Kit (Invent Biotechnologies, Inc., Plymouth, MN, USA). Western blots were probed with an anti-ZHX2 antibody (Proteintech Group, Inc., London, UK) followed by incubation with an HRP-conjugated secondary antibody. Protein bands were detected using the chromogenic substrate Ez West Blue W (Atto Corp., Tokyo, Japan).

### Statistics

Peason’s correlation coefficient test was used to verify the relationship between the level of protein expression and the severity of gene alteration. The significance level of P < 0.05 was used.

## Results

### Ubiquitination of ZHX2

**Figure 1:**
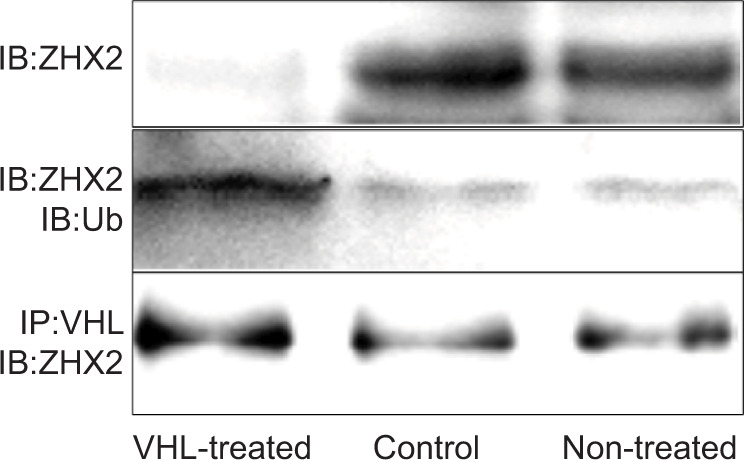
Immunoprecipitation and Western blotting study using anti-ZHX2 antibody for Scl+ MNSCs. MNSC-derived cells before and after treatment with VHL-expressing adenovirus vector. Upper, Immunoblotting with anti-ZHX2 antibody (Upper). Middle, Immunoprecipitation with anti-ZHX2 antibody followed by immunoblotting with anti-ubiquitin antibody. Lower, Immunoprecipitation with anti-VHL antibody followed by immunoblotting with anti-ZHX2 antibody.

We asked whether or not pVHL mediates ZHX2 ubiquitination. We found that the compound containing pVHL has E3 ubiquitin ligase activity and mediates ZHX2 ubiquitination. This result suggested that pVHL mediates ZHX2 ubiquitination (Figure 1). This result then indicated that ubiquitination of ZHX2 was associated with hemangioblastoma tumorigenesis.

### Protein expression of ZHX2 analyzed by Western blotting

**Figure 2:**
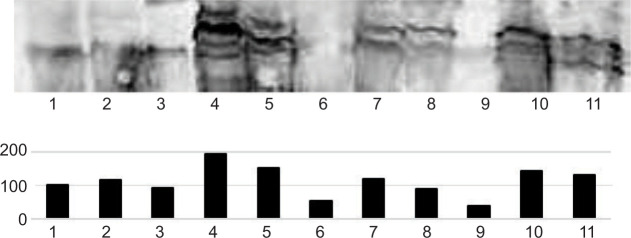
Western blot analysis for ZHX2. Top, raw data of Western blot analysis. Bottom, Histogram of Western blot analysis. Lane 1, Sporadic hemangioblastoma (HB); Lane 2, Sporadic HB; Lane 3, Sporadic HB; Lane 4, VHL-HB; Lane 5, VHL-HB; Lane 6, Sporadic HB; Lane 7, VHL-HB; Lane 8, VHL-HB; Lane 9, Sporadic HB; Lane 10, VHL-HB; Lane 11, VHL-HB.

Protein expression levels of ZHX2 on cultured Scl+ hemangioblast-like cells and hemangioblastoma tissues were analyzed by Western blotting, with the following results: expression of ZHX2 was shown in Scl+ hemangioblast-like cells, while cells treated with VHL-expressing adenovirus vector lost expression of ZHX2. The expression of ZHX2 in CNS hemangioblastoma tissues was shown. High expression (+++) was set at WB histogram >150; intermediate expression (++) at 150 to 90; low expression (+) at 90 to 30; negative expression (−) at <30. The results showed different levels of ZHX2 expression: high in two cases, medium in four, low in four cases, and negative in one (Figures 1 and 2; Table 1).

**Table 1:** Characteristics of VHL-related and sporadic CNS-HB.

Case No.	CNS-HB type	age/sex	CNS-HB location	VHL mutation	WB	IHC
1	VHL	28M	Cerebellum	p.Leu158Val	++	N/A
2	VHL	57M	Cerebellum	p.Leu158Val	+	N/A
3	VHL	46M	Cerebellum	Exon1,2,3 del	+++	+++
4	VHL	47M	Cerebellum	Exon 3 del	+	N/A
6	VHL	24M	Cerebellum	Exon 2 del	+++	++
7	VHL	28M	Cerebellum	p.Arg157Gln	++	N/A
8	VHL	27M	Cerebellum	p.Pro81Ser	++	++
9	VHL	26F	Cerebellum	Exon 1,2,3 del	N/A	+
10	VHL	31M	Cerebellum	p.Ser80Ile	N/A	+++
11	VHL	54M	Cerebellum	p.Cys161Thr	N/A	++
12	VHL	32M	Cerebellum	exon 1,2,3 del	N/A	-
13	VHL	9F	Cerebellum	p.Arg157X	N/A	-
14	VHL	38M	Cerebellum	p.76Phe del	N/A	-
5	Sporadic	26M	Cerebellum	No mutation	+	N/A
15	Sporadic	28F	Cerebellum	No mutation	+	-
16	Sporadic	37F	Cervical spinal cord	Spile site c.340-1	-	N/A
17	Sporadic	52M	Cervical spinal cord	c.450del	+	N/A
18	Sporadic	43M	Cervical spinal cord	No mutation	N/A	-
19	Sporadic	56M	Cervical Spinal cord	No mutation	N/A	-
20	Normal brain	61M	Cerebrum	N/A	N/A	-

CNS-HB, central nervous system hemangioblastoma; M, male; F, female; N/A, not available; WB, Western blot analysis; IHC, immunohistochemical analysis; +++, high-level expression; ++, medium; level expression; +, low-level expression; −, negative expression.

### Immunohistochemical analysis of ZHX2 expression in CNS-HB tissues

**Figure 3:**
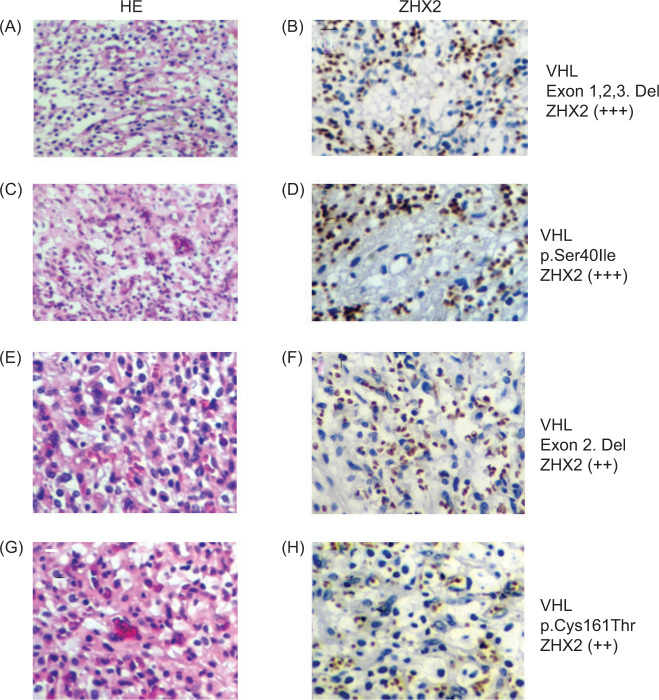
Pathohistological analysis. A, C, E, G, hematoxylin-eosin (HE) stain. B, D, F, H, ZHX2-immunohistochemical stain. ++, high level expression of ZHX2; +, medium level expression of ZHX2. All cases are VHL-related central nervous system hemangioblastomas (CNS-HBs).

ZHX2 expression was mainly localized in the nuclei of stromal cells in CNS-HB. High expression (+++) was defined as ZHX2-positive >60% of stromal cells, intermediate expression (++) as ZHX2-positive 60–30% of cells, low expression (+) as ZHX2-positive 30–5% of cells, and negative expression as ZHX2-positive <5% of cells. Immunohistochemical analysis revealed variable levels of ZHX2 expression in CHS-HB: high in two cases, intermediate in three cases, low in one, and negative in seven. Conversely, ZHX2 expression was not detected in the normal cerebrum. Among the cases showing immunohistochemical positivity for ZHX2, five were associated with VHL-associated CNS-HB, of which three were VHL-associated and four were sporadic (Figures 2 and 3; Table 1).

### Germline and somatic mutations in CNS hemangioblastomas

Germline and somatic mutations in the VHL gene in CNS hemangioblastomas (HBs) were analyzed. All 14 VHL-associated HBs showed mutations in the VHL gene, including eight missense mutations, five exon deletions and one amino acid deletion. In contrast, two out of six sporadic CNS HBs showed VHL gene mutations, including one nucleotide deletion resulting in a frameshift and one splice site mutation (Table 1).

### Association between VHL gene mutation and ZHX2 expression

Western blot analysis revealed high levels of ZHX2 expression in two VHL-related CNS-HB cases with exon deletions. Moderate expression of ZHX2 was observed in three VHL-related CNS-HB cases with missense mutations. ZHX2 showed low or weak expression in two VHL-related HBs with missense mutation and exon deletion, four sporadic CNS-HBs with splice site mutation (1), single nucleotide deletion (1), and four cases with no data available. Immunohistochemical analysis showed high positivity in two VHL-associated HBs with exon deletion and missense mutation. Moderate positivity was observed in three VHL-associated HBs with exon deletion (1) and missense mutation (2). Weak positivity was observed in one VHL-related HB with exon deletion. In contrast, immunohistochemical negativity was observed in three VHL-associated CNS-HBs with exon deletion and missense mutation (2) and three sporadic CNS-HBs without mutation in the VHL gene (Table 1). These results suggest that ZHX2 expression is present in VHL-deleted CNS-HBs, especially in cases with larger alterations in the VHL gene. The relationship between VHL gene mutation and ZHX2 expression in CNS-HBs revealed that the severity of VHL gene alteration was significantly correlated with the level of immunoblotting (P < 0.05), while the severity of VHL gene alteration was not significantly correlated with immunohistochemical positivity (P = 0.1).

## Discussion

Previously, it has been suggested that the neoplastic cell of origin in CNS hemangioblastoma is the mesoderm-derived embryonic hemangioblast (19), and that stromal cells of hemangioblastoma have mesenchymal stem cell-derived vascular progenitor properties (2120). Recently, we isolated Scl+ hemangioblast-like cells from CNS hemangioblastoma and used these cells to analyze the VHL-ZHX2 signaling pathway in CNS hemangioblastoma. In this study, after the VHL-expressing adenovirus vector was transferred into Scl+hemangioblast-like cells, immunoprecipitation between pVHL and ZHX2 was detected, and ZHX2 was ubiquitinated in these cells. Thus, this result suggested that ZXH2 is degraded after ubiquitination (Figure 1). This result was supported by another result that the expression of ZHX2 was detected in CNS hemangioblastoma tissues depending on the type of VHL gene mutation, and this result also suggested that the VHL-mutant hemangioblastoma cells promote the expression of ZHX2.

The VHL-HIF signaling pathway is considered to be the primary axis of VHL disease and its downstream pathways. Subsequently, HIF-VEGF-VEGFR2, HIF-EPO, HIF-CAIX, HIF-SDF1β-CXCR4, HIF-EphB4/EphrinB2, and HIF-Notch/Dll4 are thought to play critical roles in angiogenesis in hemangioblastoma (5 ,9, 21–25). VHL physically interacts with HIF, which is necessary for its oxygen-dependent degradation (26, 27). Recently, the JAK-STAT signaling pathway we proposed has been implicated in various tumor-associated processes, including tumorigenesis, cell proliferation, stemness maintenance, angiogenesis, invasion, metastasis and immune regulation (28,–35). Conversely, the VHL substrate transcription factor ZHX2 has been identified as an oncogenic driver in VHL-deficient clear cell renal cell carcinoma (ccRCC). ZHX2 expression was identified exclusively in the nuclei of ccRCC cells, with VHL WT ccRCC showing less pronounced expression than the VHL frameshift mutant. These findings suggest that ZHX2 plays a specific role in VHL-deficient ccRCC and not in VHL-WT ccRCC (12). Thus, the VHL-ZHX2 signaling pathway appears to play an important role in VHL-deficient ccRCC.

**Figure 4:**
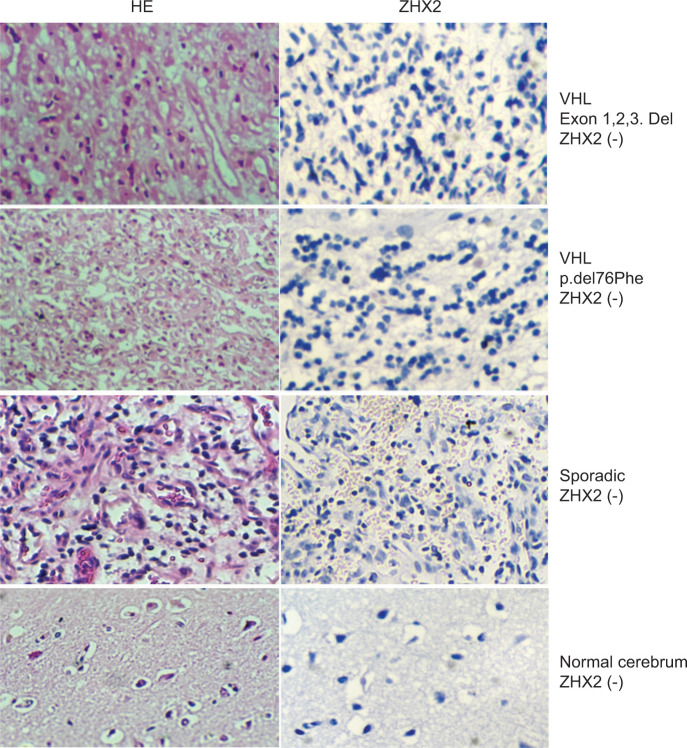
Pathohistological analysis. A, C, E, G, hematoxylin-eosin (HE) stain. B, D, F, H, ZHX2 immunohistochemical staining. (−) Negative expression. Del, deletion; all cases are negative.

In this study, ZHX2 expression was detected in VHL-related and sporadic CNS-HB using Western blot analysis and immunohistochemistry. ZHX2 localized predominantly in the nuclei of stromal cells in CNS-HBs, similar to VHL-deficient ccRCC (12). In addition, VHL-related CNS-HB shows higher ZHX2 expression than sporadic CNS-HB. Furthermore, ZHX2 expression was more pronounced in VHL-related CNS-HB with larger alterations in the VHL gene than in those with small alterations, similar to VHL-deficient ccRCC (12). In other words, ZHX2 expression was correlated to the severity of the VHL gene alteration in CNS-HB. These results suggest that VHL inactivation promotes ZHX2 and its downstream factors. ZHX2 and HIF cooperate to drive cancer cell proliferation in triple-negative breast cancer (36). In CNS-HB, ZHX2 may function alongside HIF in proliferation and maintenance. The VHL-ZHX2 signaling pathway could potentially contribute to cell proliferation and angiogenesis in CNS-HB, serving as an alternative pathway to complement the established VHL-HIF and VHL-JAK-STAT signaling pathways. Consequently, the VHL-ZHX2 signaling pathway emerges as a plausible therapeutic target for hemangioblastoma.

## Conclusion

ZHX2 expression was detected in the nuclei of stromal cells in both VHL-related and sporadic CNS-HB. As in VHL-deficient ccRCC, ZHX2 expression was significantly more pronounced in VHL-related CNS-HBs with larger alterations compared to those with smaller alterations in the VHL gene. In other words, ZHX2 expression was correlated with the severity of VHL gene alteration in CNS-HB, suggesting a potential role in tumorigenesis and proliferation. Thus, the VHL-ZHX2 pathway could potentially contribute to cell proliferation and angiogenesis in CNS-HB, serving as an alternative pathway to complement the established VHL-HIF and VHL-JAK-STAT pathways. Thus, the VHL-ZHX2 pathway emerges as a plausible therapeutic target for hemangioblastoma.
